# Trends in cause and place of death for children in Portugal (a European country with no Paediatric palliative care) during 1987–2011: a population-based study

**DOI:** 10.1186/s12887-017-0970-1

**Published:** 2017-12-22

**Authors:** Ana Forjaz de Lacerda, Barbara Gomes

**Affiliations:** 1 King’s College London, Cicely Saunders Institute of Palliative Care, Policy and Rehabilitation, Bessemer Road, London, SE5 9PJ UK; 2 Portuguese Institute of Oncology – Lisbon Centre, Paediatrics Department, Lisboa, Portugal; 3 University of Coimbra, Faculty of Medicine, Coimbra, Portugal

**Keywords:** Child, Epidemiology, Health services, Mortality, Palliative care

## Abstract

**Background:**

Children and adolescents dying from complex chronic conditions require paediatric palliative care. One aim of palliative care is to enable a home death if desired and well supported. However, there is little data to inform care, particularly from countries without paediatric palliative care, which constitute the majority worldwide.

**Methods:**

This is an epidemiological study analysing death certificate data of decedents aged between 0 and 17 years in Portugal, a developed Western European country without recognised provision of paediatric palliative care, from 1987 to 2011. We analysed death certificate data on cause and place of death; the main outcome measure was home death. Complex chronic conditions included cancer, cardiovascular, neuromuscular, congenital/genetic, respiratory, metabolic, gastro-intestinal, renal, and haematology/immunodeficiency conditions. Multivariate analysis determined factors associated with home death in these conditions.

**Results:**

Annual deaths decreased from 3268 to 572. Of 38,870 deaths, 10,571 were caused by complex chronic conditions, their overall proportion increasing from 23.7% to 33.4% (22.4% to 45.4% above age 1-year). For these children, median age of death increased from 0.5 to 4.32-years; 19.4% of deaths occurred at home, declining from 35.6% to 11.5%; factors associated with home death were year of death (adjusted odds ratio 0.89, 95% confidence interval 0.89–0.90), age of death (6–10 year-olds 21.46, 16.42–28.04, reference neonates), semester of death (October–March 1.18, 1.05–1.32, reference April–September), and cause of death (neuromuscular diseases 1.59, 1.37–1.84, reference cancer), with wide regional variation.

**Conclusions:**

This first trend analysis of paediatric deaths in Portugal (an European country without paediatric palliative care) shows that palliative care needs are increasing. Children are surviving longer and, in contrast with countries where paediatric palliative care is thriving, there is a long-term trend of dying in hospital instead of at home. Age, diagnosis, season and region are associated with home death, and should be considered when planning services to support families choosing this option. Priorities should address needs of the youngest children, those with cancer, neuromuscular and cardiovascular conditions, as well as inequities related to place of residence.

**Electronic supplementary material:**

The online version of this article (doi: 10.1186/s12887-017-0970-1) contains supplementary material, which is available to authorized users.

## Background

Due to public health improvements and medical advances, paediatric deaths are rare in developed countries [1–3]. However, children should not be forgotten when integrating palliative care (PC) in the healthcare continuum, as recently recommended by the World Health Organization [4]. Worldwide, only 5.7% of countries provide well-developed paediatric palliative care (PPC); 66% have no reported activity, compared to 32% for adults [5, 6]. Hindering PPC development is a non-uniform definition of need. A step forward was taken with a definition of “complex chronic conditions” (CCCs), considering function, quality of life and service needs - “any medical condition that can be reasonably expected to last at least 12 months (unless death intervenes) and to involve either several different organ systems or one organ system severely enough to require specialty pediatric care and probably some period of hospitalization in a tertiary care center”(pg.206) [7].

PC aims, among other goals, to enable a home death (HD) if desired and well supported [1, 8, 9]. While systematic reviews show that most adults would prefer to die at home [10], paediatric evidence is scarcer and more heterogeneous [11]. However, it seems that the opportunity to plan place of death (PoD), reflecting family choice, promotes parental preparedness and comfort [12]. Caring for and enabling a child to die at home increases parental adaptation to loss [13, 14], while decreasing the burden on healthcare providers [14, 15].

Both cause and PoD are essential for informing service development but are understudied in Paediatrics. Most research originates from culturally similar countries with mature PPC services [2, 7, 16–25] showing that most families prefer care and death at home when support is available, and that the HD proportion is high and increasing.

There is great need for epidemiological evidence from different settings. Portugal is a Western European developed country with a publicly-funded healthcare system (with income-modulated fees), praised for its achievements in childhood health indicators [26]. However, while adult PC availability is considered to be generalised (group 3b), no paediatric provision is yet recognised [5, 6]. Therefore, Portugal represents the ideal setting for our aim: to examine the epidemiological situation of paediatric deaths in a country without PPC, describing trends in cause and PoD and factors related to HD in CCCs (disease-related, socio-demographic and environmental).

## Methods

This is a cross-sectional epidemiological population-based study of mortality data, examining death certificates for individuals deceased in Portugal from 1987 to 2011, before their eighteenth birthday (legal paediatric age-limit). The National Institute of Statistics (NIS) provided a dataset free-of-charge with individual anonymised death certificate information; the country’s death certificate data are considered to be of medium-high quality [27]. Data were analysed in IBM SPSS v21® and Excel:Mac2011 v14©. STROBE guidelines and methodology were adhered to.

*Cause of death* (1987–2001 International Classification of Diseases, 9th revision - ICD-9; 2002–2011 ICD-10) was recoded in three major groups: CCCs (Additional file 1: Table S1) [2, 18], other medical causes (OMCs), and trauma.

In Portuguese death certificates, *PoD* is recorded in three categories: “domicile*”* (any non-public non-clinical place), “hospital/clinic*”* (any clinical facility), and “other place*”* (any public space). Since there are no paediatric hospices, it can be assumed that “domicile” represents home, and “hospital/clinic” represents hospital. For CCCs, the PoD analysis focused on home versus elsewhere (hospital/other place).

*Gender*, *nationality*, *place of residence*, *age*, *cause* and *date of death* (weekday, month, trimester, semester, year) were examined as potential explanatory variables of PoD. *Nationality* was thought to be of relevance since there is a large number of resident immigrant children from African Portuguese speaking countries (former Portuguese colonies). *Age* was non-normally distributed and analysed non-parametrically. For infants, there was additional data on *parents’ age*, *education* and *working status*. *Place of residence* was the only variable available to inform on socio-economic status; it was used as a proxy, transformed (according to 2011 area level tools provided by the NIS) into *urbanisation level* (high, medium, and low), *population density* (quintiles) and *bed ratio* (number of beds in health establishments per 1000 inhabitants, above or below national average).

Three variables with high levels of missing data were excluded (*urbanisation level, mothers’ working status,* and *father’s educational level*); multiple imputation was deemed inappropriate as it would lead to an unacceptable level of assumptions (missing data in 31.4–66.7% of cases).

To control for differences in age and gender distribution over time, we calculated crude and standardised percentages of deaths by PoD using direct standardisation (1987’s decedent population as standard), as well as the infant mortality rate, using NIS population data. For CCCs, bivariate analyses explored associations between HD and potential explanatory variables; those with significant association (*p* < 0.05) were entered into a multivariate analysis (MVA), conducted on complete cases. Logistic regression models were run using backwards-stepwise likelihood-ratio selection of variables; goodness-of-fit was evaluated by Wald statistics and the model’s λ^2^, Nagelkerke R^2^ and classification table.

For clinical usefulness and comparability [2, 21] we conducted sub-analyses for decedents under and above age 1-year. For infants, the model retaining sub-region was rejected for having high standard error in Wald statistics (probably due to very small numbers of HD in most sub-regions).

## Results

In this 25-year period, 38,870 deaths of 0–17 year-olds (yo) were registered in Portugal, representing 1.5% of all deaths (decreasing from 3.4% in 1987 to 0.6% in 2011). Annual paediatric deaths decreased by 82.5% (3286 to 572) and the infant mortality rate by 76% (from 9.9 to 2.4).

### Cause of death

**Fig. 1:**
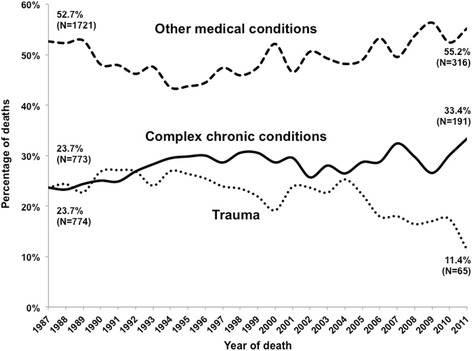
Cause of death of 0–17 year-olds in Portugal (1987–2011, *N* = 38,870). *Footnote*: Percentages may not add to 100 due to rounding. λ^2^ for trend (complex chronic conditions vs. others) 72.722, 1df, *p* < 0.001

CCCs caused 27.2% of deaths, increasing from 23.7 to 33.4% (Fig. 1); this increase was more pronounced beyond infancy, from 22.4 to 45.4%. OMCs caused 49.1% and trauma 23.7% of all deaths.

Of CCCs’ decedents 55.0% were male and 50.6% were infants (Table 1). Over the study period age at death gradually increased: median age of death increased from 0.50 years (interquartile range 0.01–6.79) in 1987 to 4.32 years (0.10–10.47) in 2011. This was mostly due to a decline in the proportion of infants’ deaths (from 56.3 to 36.1% of CCCs’ deaths) and an increase in adolescents’ 11-17yo (16.8 to 24.1%).

**Table 1:** Paediatric deaths from complex chronic conditions in Portugal (1987–2011) – demographics, cause and place of death

Variable	Category	All CCCs	CCC categories								
			Cancer	Neuromuscular	Cardiovascular	Respiratory	Renal	Gastrointestinal	Haem. & Immun.	Metabolic	Other
N(%)		10,571(100)	2811(26.6)	2128(20.1)	2600(24.6)	419(4.0)	195(1.8)	242(2.3)	181(1.7)	284(2.7)	1711(16.2)
Gender	Male	5814(55.0)	1558(55.4)	1153(54.2)	1433(55.1)	249(59.4)	128(65.6)	137(56.6)	111(61.3)	147(51.8)	898(52.5)
	Female	4756(45.0)	1253(44.6)	975(45.8)	1167(44.9)	170(40.6)	67(34.4)	105(43.4)	70(38.7)	137(48.2)	812(47.5)
Nationality	Portuguese	7078(67.0)	2111(75.1)	1475(69.3)	1761(67.7)	286(68.3)	97(49.7)	178(73.6)	139(76.8)	199(70.1)	832(48.6)
	Other	2102(19.9)	697(24.8)	414(19.5)	492(18.9)	94(22.4)	15(7.7)	26(10.7)	42(23.2)	85(29.9)	237(13.9)
	Missing	1391(13.2)	3(0.1)	239(11.2)	347(13.3)	39(9.3)	83(42.6)	38(15.7)	0(0.0)	0(0.0)	642(37.5)
Age of death	Median, years (IQR)	0.94 (0.04–8.94)	9.86 (5.16–14.73)	2.00 (0.14–10.89)	0.18 (0.02–0.96)	0.12 (0.00–0.64)	0.00 (0.00–0.21)	0.22 (0.03–1.20)	2.17 (0.65–10.77)	1.78 (0.25–7.94)	0.01 (0.00–0.25)
	0–27 days	3055(28.9)	20(0.7)	449(21.1)	1005(38.7)	193(46.1)	135(69.2)	88(36.4)	0(0.0)	38(13.4)	1127(65.9)
	28–364 days	2289(21.7)	111(3.9)	457(21.5)	961(37.0)	138(32.9)	22(11.3)	87(36.0)	67(37.0)	91(32.0)	355(20.7)
	1–5 years	1855(17.5)	708(25.2)	405(19.0)	413(15.9)	30(7.2)	17(8.7)	38(15.7)	49(27.1)	71(25.0)	124(7.2)
	6–10 years	1259(11.9)	733(26.1)	290(13.6)	102(3.9)	18(4.3)	3(1.5)	13(5.4)	21(11.6)	34(12.0)	45(2.6)
	11–14 years	1046(9.9)	586(20.8)	280(13.2)	64(2.5)	21(5.0)	11(5.6)	9(3.7)	13(7.2)	30(10.6)	32(1.9)
	15–17 years	1067(10.1)	653(23.2)	247(11.6)	55(2.1)	19(4.5)	7(3.6)	7(2.9)	31(17.1)	20(7.0)	28(1.6)
	<1 year	5344(50.6)	131(4.7)	906(42.6)	1966(75.6)	331(79.0)	157(80.5)	175(72.3)	67(37.0)	129(45.4)	1482(86.6)
	≥1 year	5227(49.4)	2680(95.3)	1222(57.4)	634(24.4)	88(21.0)	38(19.5)	67(27.7)	114(63.0)	155(54.6)	229(13.4)
Place of death	Home	2052(19.4)	794(28.2)	623(29.3)	333(12.8)	21(5.0)	24(12.3)	23(9.5)	21(11.6)	55(19.4)	158(9.2)
	Hospital	8383(79.3)	1997(71.0)	1452(68.2)	2230(85.8)	397(94.7)	171(87.7)	218(90.1)	159(87.8)	226(79.6)	1533(89.6)
	Other	136(1.3)	20(0.7)	53(2.5)	37(1.4)	1(0.2)	0(0.0)	1(0.4)	1(0.6)	3(1.1)	20(1.2)

*Haem. & Immun* Haematology and Immunodeficiency, *IQR* interquartile range, *Other* other congenital and genetic conditions. Except for “N” and “Median age of death”, values in parenthesis refer to percentages within categories in each column. Percentages may not add up to 100 due to rounding. The high percentages of missing data for nationality in children dying from renal and other congenital / genetic causes are probably due to death occurring soon after birth, before civil registration. Gender information was missing only for one child (a neonate with other congenital / genetic condition), and there were no missing data for age and place of death

Cancer was the leading cause of death from CCCs (Table 1), increasing from 24.6 to 38.2% of CCCs’ deaths over the time period. Solid tumours caused more CCC deaths (16.1%) than haematological malignancies (10.5%).

In infants, the predominant CCC diagnoses were cardiovascular, other congenital/genetic, and neuromuscular; in neonates, congenital/genetic conditions prevailed. Beyond infancy, cancer caused half the CCCs’ deaths; neuromuscular and cardiovascular diseases caused another third. Median age of death (Table 1) varied by diagnosis, from 0.00 (renal conditions) to 9.86 years (cancer).

### Place of death

Overall, hospital was the most common PoD, increasing from 65.8 to 79.7% (*p* < 0.001; Additional file 2: Figure S1). This trend was not explained by demographic changes, as it maintained with age- and gender- standardised proportions. Deaths in public places were mostly due to trauma (67.4%).

**Fig. 2:**
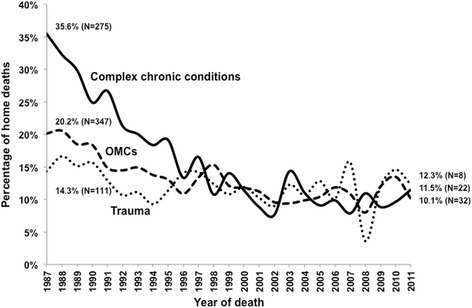
Home deaths of 0–17 year-olds in Portugal (1987–2011, *N* = 38,870) by cause of death. *Footnote*: λ^2^ for trend (home vs. elsewhere): Complex Chronic Conditions 406.900, 1df, *p* < 0.001; Other Medical Causes (OMCs) 153.067, 1df, *p* < 0.001; Trauma 13.746, 1df, *p* < 0.001

Only 15.6% of deaths occurred at home, more commonly in CCCs (19.4%) than OMCs (14.8%) or trauma (12.8%). A significant trend towards death away from home was most evident for CCCs (Fig. 2 and Additional file 3: Table S2); despite yearly fluctuations, the last 10 years showed a stabilisation of HDs around 10% for CCCs, and 11% for OMCs and trauma.

#### Home death in complex chronic conditions

Over 25 years, HD in CCCs decreased from 35.6 to 11.5%, the lowest being 7.6% in 2002 (Fig. 2). This trend was also not explained by demographic changes (Additional file 4: Figure S2).

The decrease was more marked in infants and in cancer (Additional files 5 and 6: Figures S3 and S4), and it was not equal across the country. It was more striking in the central (50.9 to 16.1%), and northern (44.0 to 15.0%) than in the capital region (15.8 to 7.0%).

In the bivariate analysis (Additional file 7: Table S3; all differences statistically significant to at least 0.05), the occurrence of HD differed by gender (females 20.3%, males 18.7%), and nationality (Portuguese 25.5%, foreigners 10.1%). Infants (9.7%) died less often at home than older children (29.4%). There were wide regional variations, from a lowest of 5.1% in the southern littoral to 58.5% in the central interior (Additional file 8: Figure S5). The relation between HD and population density was non-linear; HD ranged from 14.2% in the most densely populated quintile to 34.7% in the second least populated. Areas with below-average bed ratios had more HDs (24.1%) than those above-average (9.9%). More HDs happened between October–March (20.7%) than April–September (18.0%); there was no significant association with other time variables. The occurrence of HD varied between 5.0% in respiratory and 29.3% in neuromuscular diseases. Although deaths from haematological malignancies happened less often at home (26.5%) than those from solid tumours (29.4%), this difference was not significant (*p* = 0.096). For all diagnoses, HD was more frequent above age 1-year (11.4% in respiratory to 39.3% in neuromuscular conditions) than in infancy (3.3% in respiratory conditions to 20.6% in cancer). Children with non-cancer conditions died less often at home (16.2% versus 28.2% in cancer). For infants (Additional file 9: Table S4), HD was more common when the mother had no/basic education (10.9%, 5.0% when more educated). Both parents’ age and fathers’ working status were not statistically significant.

The MVA model for 0-17yo retained five variables as independently associated with HD: year, age, diagnosis, semester, and sub-region (Table 2). The odds of HD decreased by 11% annually. Age influence was non-linear: HD odds increased from neonates (reference) to 6-10yo, decreasing thereafter. Compared to cancer, the highest HD odds were found in neuromuscular and the lowest in respiratory conditions. Deaths in October–March showed higher HD odds than in April–September. HD odds were lowest in Greater Lisbon (capital) and the southern littoral (Algarve and Alentejo Litoral) and highest in a central interior mountainous area (Serra da Estrela).

**Table 2:** Factors associated with paediatric home deaths from complex chronic conditions in Portugal (1987–2011)

Variable	Category	Home death	Unadjusted odds ratios for home death	Adjusted odds ratios for home death^a^			
		%	N	OR	95% CI	AOR	95% CI
Year of death	1987	35.6	767	1		1	
	Successive years	NA	NA	0.92	0.91–0.93	0.89	0.89–0.90
Age	0–27 days	3.3	3039	1		1	
	28–364 days	18.2	2259	6.48	5.17–8.11	8.67	6.81–11.04
	1–5 years	27.2	1818	10.70	8.56–13.38	14.26	11.11–18.31
	6–10 years	34.8	1236	15.17	12.05–19.09	21.46	16.42–28.04
	11–14 years	30.4	1032	12.55	9.86–15.92	16.23	12.34–21.34
	15–17 years	27.9	1056	11.18	8.80–14.20	14.99	11.35–19.79
Cause of death	Cancer	29.0	2739	1		1	
	Neuromuscular	29.3	2116	1.05	0.93–1.19	1.59	1.37–1.84
	Cardiovascular	12.9	2569	0.37	0.32–0.43	0.67	0.56–0.81
	Respiratory	5.0	418	0.13	0.09–0.21	0.29	0.18–0.47
	Renal	12.3	195	0.36	0.23–0.55	1.15	0.68–1.94
	Gastro-intestinal	9.7	237	0.27	0.17–0.41	0.40	0.24–0.64
	Haematology & Immunodeficiency	11.7	18	0.33	0.21–0.53	0.59	0.36–0.98
	Metabolic	19.4	283	0.61	0.45–0.83	0.88	0.62–1.24
	Other congenital & genetic	9.3	1703	0.26	0.22–0.31	0.87	0.69–1.09
Semester of death	Spring-summer	18.1	4977	1		1	
	Autumn-winter	21.0	5463	1.19	1.08–1.31	1.18	1.05–1.32
Sub-region	Greater Lisbon	8.7	1916	1		1	
	Minho-Lima	27.5	240	4.00	2.89–5.53	4.46	3.10–6.41
	Cávado	22.4	478	3.04	2.33–3.97	3.66	2.73–4.91
	Ave	22.0	563	2.98	2.31–3.84	3.31	2.50–4.37
	Greater Porto	19.1	1221	2.49	2.01–3.08	2.91	2.31–3.67
	Tâmega	27.8	709	4.06	3.23–5.10	5.34	4.14–6.87
	Entre Douro e Vouga	25.4	276	3.58	2.62–4.91	4.43	3.13–6.26
	Douro	38.9	262	6.72	5.00–9.03	7.60	5.44–10.62
	Alto Trás os Montes	36.1	241	5.96	4.38–8.10	6.85	4.81–9.77
	Baixo Vouga	27.0	370	3.91	2.95–5.16	4.13	3.04–5.62
	Baixo Mondego	30.5	272	4.63	3.42–6.27	5.08	3.62–7.14
	Pinhal Litoral	31.2	215	4.77	3.43–6.64	5.45	3.76–7.88
	Pinhal Interior Norte	40.8	120	7.28	4.89–10.83	8.72	5.51–13.79
	Dão-Lafões	31.6	304	4.87	3.64–6.50	5.76	4.16–7.97
	Pinhal Interior Sul	28.6	35	4.22	1.99–8.93	4.05	1.76–9.30
	Serra da Estrela	58.5	53	14.86	8.41–26.24	24.00	11.80–48.80
	Beira Interior Norte	35.2	105	5.74	3.73–8.83	6.43	3.93–10.50
	Beira Interior Sul	25.8	62	3.67	2.03–6.62	4.25	2.14–8.45
	Cova da Beira	30.8	78	4.69	2.82–7.78	4.85	2.71–8.67
	Oeste	15.7	281	1.96	1.37–2.80	1.98	1.34–2.91
	Médio Tejo	24.3	251	3.39	2.44–4.71	3.25	2.26–4.67
	Península de Setúbal	10.0	607	1.18	0.87–1.60	1.08	0.78–1.50
	Lezíria do Tejo	13.4	164	1.63	1.01–2.63	1.49	0.89–2.48
	Alentejo Litoral	5.1	78	0.57	0.21–1.58	0.57	0.20–1.64
	Alto Alentejo	13.3	113	1.61	0.92–2.84	1.50	0.81–2.75
	Alentejo Central	19.4	134	2.54	1.61–4.01	2.79	1.68–4.63
	Baixo Alentejo	18.0	128	2.31	1.43–3.73	2.05	1.23–3.43
	Algarve	6.2	372	0.70	0.44–1.09	0.77	0.48–1.23
	Azores	14.8	413	1.83	1.33–2.50	1.90	1.35–2.67
	Madeira	10.8	379	1.28	0.89–1.84	1.27	0.87–1.87

*N* = 10,440. NA – not applicable; OR – odds ratio. Categories with an OR/AOR of “1” are reference categories. *p*-values <0.001 for all variables except semester (*p* = 0.004)

^a^Model λ^2^ 2482.058, 44df, p < 0.001; Nagelkerke R^2^ 0.337; correct classification of place of death 83.5% (home 34.2%, elsewhere 95.5%)

The infants’ model retained six variables (Table 3): year, age, diagnosis, semester, population density and bed ratio. As for 0-17yo, HD odds were higher in 1987, decreasing thereafter 14% annually; dissimilarly, HD odds were higher in cancer and lower in both respiratory and gastro-intestinal conditions. Increased HD odds were associated with below-average bed availability, as well as deaths in October–March. As in bivariate analysis, the association between HD and population density was non-linear.

**Table 3:** Factors associated with home death in infants and older children from complex chronic conditions in Portugal (1987–2011)

Variable	Category	<1 year-olds							1–17 year-olds				
		HD		OR for HD		AOR for HD^a^		HD		OR for HD		AOR for HD^b^	
		%	N	OR	95%CI	AOR	95%CI	%	N	OR	95%CI	AOR	95%CI
Year of death	1987	21.7	433	1		1		53.6	334	1		1	
	Successive years	NA		0.88	0.86–0.90	0.86	0.84–0.88	NA		0.91	0.90–0.92	0.90	0.89–0.91
Age bands	0–27 days	3.3	3039	1		1							
	28–364 days	18.2	2259	6.48	5.17–8.11	8.53	6.64–10.97						
	1–5 years							27.2	1818	1		1	
	6–10 years							34.8	1236	1.42	1.21–1.66	1.45	1.22–1.74
	11–14 years							30.4	1032	1.17	0.99–1.39	1.10	0.91–1.32
	15–17 years							27.9	1056	1.04	0.88–1.24	1.01	0.84–1.23
Cause of death	Cancer	20.3	128	1		1		29.4	2611	1		1	
	Neuromuscular	15.7	898	0.72	0.46–1.14	0.95	0.56–1.59	39.2	1218	1.61	1.40–1.86	1.70	1.45–2.00
	Cardiovascular	11.0	1943	0.47	0.30–0.74	0.59	0.36–0.98	19.0	626	0.58	0.46–0.72	0.50	0.39–0.63
	Respiratory	3.3	330	0.13	0.06–0.28	0.19	0.09–0.42	11.4	88	0.32	0.17–0.62	0.31	0.15–0.62
	Renal	6.4	157	0.26	0.12–0.57	0.88	0.37–2.04	36.8	38	1.46	0.75–2.83	1.13	0.55–2.33
	Gastrointestinal	5.2	172	0.21	0.09–0.46	0.19	0.08–0.45	21.5	65	0.66	0.36–1.19	0.56	0.30–1.08
	Haem. & Imunn.	7.5	67	0.31	0.11–0.85	0.42	0.14–1.24	14.2	113	0.41	0.24–0.70	0.60	0.33–1.06
	Metabolic	7.0	129	0.29	0.13–0.64	0.39	0.16–0.93	29.9	154	1.05	0.74–1.50	1.09	0.74–1.61
	Other	6.0	1474	0.24	0.15–0.39	0.52	0.30–0.89	30.6	229	1.10	0.82–1.47	1.17	0.85–1.62
Semester of death	April–September	8.3	2592	1		1							
	October–March	11.0	2706	1.36	1.13–1.63	1.41	1.15–1.74						
Population density	5th quintile	6.1	2654	1		1							
	4th quintile	13.3	1079	2.37	1.87–3.00	1.76	1.33–2.33						
	3rd quintile	10.4	857	1.78	1.36–2.34	1.60	1.18–2.18						
	2nd quintile	21.3	446	4.16	3.16–5.49	3.15	2.26–4.39						
	1st quintile	8.4	262	1.41	0.89–2.24	1.10	0.66–1.85						
Bed Ratio	Above average	3.5	1664	1		1							
	Below average	12.5	3634	3.87	2.93–5.12	2.99	2.17–4.13						
Sub-region	Greater Lisbon							14.6	954	1		1	
	Minho-Lima							42.0	112	4.24	2.80–6.43	3.98	2.56–6.17
	Cávado							37.0	211	3.44	2.47–4.79	3.62	2.54–5.15
	Ave							32.1	262	2.77	2.02–3.79	2.76	1.98–3.85
	Greater Porto							33.2	555	2.91	2.26–3.74	3.06	2.35–3.99
	Tâmega							41.5	325	4.17	3.13–5.54	4.68	3.46–6.34
	Entre Douro e Vouga							42.6	129	4.36	2.94–6.45	5.03	3.32–7.62
	Douro							48.6	138	5.53	3.79–8.08	5.19	3.47–7.76
	Alto Trás os Montes							50.8	122	6.06	4.07–9.02	5.49	3.59–8.41
	Baixo Vouga							41.5	195	4.17	2.96–5.84	4.05	2.84–5.78
	Baixo Mondego							42.6	148	4.35	3.00–6.31	4.26	2.87–6.32
	Pinhal Litoral							41.8	122	4.21	2.82–6.30	4.53	2.96–6.94
	Pinhal Interior Norte							58.8	68	8.38	5.00–14.02	9.60	5.50–16.77
	Dão-Lafões							42.3	149	4.30	2.96–6.23	4.15	2.80–6.16
	Pinhal Interior Sul							31.8	22	2.74	1.10–6.83	3.05	1.16–7.97
	Serra da Estrela							76.9	26	19.54	7.71–49.63	18.44	6.95–48.91
	Beira Interior Norte							50.0	58	5.86	3.40–10.11	6.24	3.48–11.18
	Beira Interior Sul							41.9	31	4.24	2.03–8.84	3.69	1.68–8.10
	Cova da Beira							42.9	35	4.40	2.20–8.80	3.62	1.73–7.60
	Oeste							21.8	147	1.63	1.06–2.51	1.60	1.02–2.51
	Médio Tejo							37.8	143	3.56	2.43–5.28	3.43	2.29–5.13
	Península de Setúbal							15.9	328	1.11	0.78–1.56	1.07	0.74–1.53
	Lezíria do Tejo							17.2	87	1.22	0.68–2.19	1.21	0.66–2.23
	Alentejo Litoral							12.1	33	0.81	0.28–2.34	0.72	0.24–2.16
	Alto Alentejo							19.7	61	1.44	0.75–2.77	1.18	0.60–2.33
	Alentejo Central							27.5	69	2.23	1.28–3.89	2.14	1.19–3.86
	Baixo Alentejo							23.6	72	1.81	1.02–3.21	1.73	0.95–3.15
	Algarve							9.9	171	0.65	0.38–1.10	0.64	0.37–1.10
	Azores							24.1	191	1.86	1.28–2.71	1.72	1.16–2.57
	Madeira							18.5	178	1.33	0.88–2.03	1.22	0.79–1.89

Children <1 year-old: *N* = 5298. Children 1–17 year-old: *N* = 5142. Haem. & Immun. – Haematology and Immunodeficiency; NA – not applicable; OR – odds ratio; Other – other congenital / genetic conditions. Categories with an OR/AOR of “1” are reference categories. The fifth population density quintile was the most populated. National bed ratio average (practiced allotment in health establishments) was 3.4 beds per 1000 inhabitants in 2011 and 3.7 in 2002; there was no change in regional categorisation between these time-points. *p*-values were 0.001 (semester) or <0.001 (all other variables)

^a^Model λ^2^ 826.310, 16df, p < 0.001; Nagelkerke R^2^ 0.307; correct classification of place of death 90.8% (home 14.5%, elsewhere 99.0%). 36/5344 (0.7%) of decedents lived abroad and 10/5344 (0.2%) had missing place of residence; these cases were excluded from the model

^b^Model λ^2^ 964.565, 41df, *p* < 0.001; Nagelkerke R^2^ 0.243; correct classification of place of death 75.8% (home 39.8%, elsewhere 91.0%). 84/5227 (1.6%) of decedents lived abroad and 1/5227 (0.0%) had missing place of residence; these cases were excluded from the model

For 1-17yo, the model retained four variables (Table 3): year, age, diagnosis, and sub-region. There was a lesser influence of year on the HD trend, with an annual 10% decrease. As in the first model, those aged 6–10, dying from neuromuscular conditions and living in the central interior had the highest HD odds; the lowest were found in the capital or southern littoral and in respiratory diseases.

## Discussion

In this trend analysis of paediatric mortality data by cause and place of death in a European country without paediatric palliative care, we found that although deaths in children and adolescents have become rare, those caused by CCCs (potentially having PPC needs) are of increasing importance. Without PPC provision, there was a long-term trend of dying away from home, more notably among CCCs. For these, MVA showed that the odds of HD were highest in the beginning of the time-series, 6-10yo, neuromuscular conditions, October–March, and central interior sub-regions. Adjusting for confounders, we found no association with gender, nationality, population density, bed availability, weekday or month of death; for infants, there was also no association with parents’ age, education or working status. This whole-population study supports reliable conclusions with implications for similar countries without PPC provision.

Overall, we found that CCCs caused 27.2% of paediatric deaths, increasing from 23.7% in 1987 to 33.4% in 2011. This represents an increase in proportion from previously reported national data using similar criteria (22.1% in the US, 1989–2003), [19] and is in line with international data showing that while paediatric mortality is decreasing, the proportion due to non-communicable illnesses is rising [28]. Our 2011 figure is comparable to 2002–2003 findings in six European countries (a study using the same CCC criteria), between 27.6 and 35.0% [2]. A more recent report from England, Scotland and Wales (2001–2010), using a newly developed ICD10 list of diagnoses states that 65–71% of decedents 1-18yo died with a chronic condition; 58% had two or more conditions [29].

As previously reported, CCCs were more frequent amid females and younger decedents [7]. However, we observed the median age of death due to CCCs increased substantially, likely explained by medical advances. This urges Paediatric Departments to prepare to deal with an increasing number of youngsters surviving through childhood with complex healthcare needs, some requiring transition to adult services. Nevertheless, in 2011 28.9% of CCCs’ decedents were infants; although decreasing (compared with 1990’s findings in Washington, 41.2%, and Ontario, 37.8%) [7, 16] this set remains critical, since it has the lowest chances of HD (1/69 in 2011). This can be partly explained by most sick newborns never being discharged after a hospital birth, challenging the realistic possibility of reverting this trend [30]. Neonatology should therefore be a greater priority for PPC, aiming to provide the best care and support for child and family wherever desired and possible.

Cancer was the main cause of death from CCCs, in higher figures (26.6%) than reported before (22%, US, 1979–1997) [18]. This may relate to the rising proportion of cancer deaths we found, in line with an increasing paediatric cancer incidence [31].

As in the US (1989–2003) [19] more than 80% of infants’ deaths were due to cardiovascular, other congenital/genetic, and neuromuscular conditions. In 1-17yo, cancer caused 51.3% of deaths, in the mid-range of recent European results (37.4–60.0% in 2002/2003); [2] alike previous reports, neuromuscular and cardiovascular conditions also prevailed [19, 20].

Compared to non-cancer CCCs, cancer patients died more often at home, a finding aligned with others [2, 19, 23]. However, differing from US results, [17, 19] we found neuromuscular conditions had the highest odds of HD. This is not unexpected, as these children usually experience a longer disease trajectory [32] which may allow for advance planning and decision-making, even in the absence of organized PPC provision. Conditions for which we found lower HD odds (e.g. respiratory) frequently rely on complex medical interventions (e.g. invasive ventilation), more prone to crisis admissions. This could also justify the difference between haematological and solid malignancies previously reported [17, 33] one we did not find; this is not easily explained, requiring further research.

While countries with PPC provision report higher and increasing trends for HD in children with CCCs, [3, 19, 34] the decreasing trend we found in Portugal means that while in 1987 1/3 of children in this group died at home, in 2011 only 1/9 did so. In the last 10 years, HD proportions have been similar for CCCs, OMCs, and trauma, highlighting the gap in care provision since expected or unexpected deaths have the same location outcome. This trend towards dying away from home, also reported for adults, [35] could not be explained by the decedents’ changing demographics. Major healthcare system reforms occurred during the 1980’s, improving accessibility to hospital services and several quality indicators; therefore, the HD decrease in acute illnesses likely represents a public health progress. However, no developments ensued in long-term and/or home-care. Accordingly, in a recent survey which found that Portuguese adults, if faced with serious terminal illness, had the lowest preference for HD (51.2%) compared to other Europeans (64.1–84.0%), the authors’ explanations were grounded on culture, religion and economy but also concern about home-care support and community resources [36]. This trend may also reflect the healthcare system and providers’ unawareness about patient-centered medicine and PC, common in the country in the last decades [37].

Previous studies have also shown associations with age and diagnosis (higher beyond infancy and in cancer), [2, 17, 19, 20] but we revealed finer diagnostic differences. As others, [2, 17, 19] we noticed wide variability between sub-regions, not explained by demographic or diagnostic profile nor PPC availability; likely explanations are topographical convenience (which may also justify more HDs in the colder months) and an insufficient number of hospital beds (although more common for adults).

Our analysis revealed a greater decreasing trend of HD in infants (annual fall 14% versus 10%), while highlighting differences in HD odds by diagnosis (lower for infants in all except cardiovascular). Interestingly, semester of death was associated with HD in infants but not in older children, warranting further research. For the 1-17yo, the decreasing trend of HD meant that in 2002–2011 the mean HD proportion was 14.7%. Meantime, in other European countries (2002/2003), this varied between 21.7–22.0% (Italy, Norway) to 31.8–50.0% (England, Wales, Belgium, Netherlands). [2] Both Italy and Norway were by then starting to develop PPC provision. In Washington State, from 1980 to 1998 the 1-17yo’s increasing trend for HD reached 43% [17].

Eight methodological limitations must be considered. First, whilst using disaggregated data from death certificates examined key variables, it did not evaluate the process of care or death [38]. As such, we cannot make considerations about quality of care or death, neither about preferences for death location. Also, we could not measure time from diagnosis to death, a factor revealed to impact on PoD since children who die less than 6 months from diagnosis are more likely to die in the hospital.[32] Secondly, although the classification systems used to code cause and PoD remained the same, there may be measurement biases due to modifications in diagnostic, reporting and coding processes over time; to the best of our knowledge, no major changes occurred. Portugal joined Eurostat (the European statistics office) in 1986 and its death certificate data are considered to be of medium-high quality (i.e. 90–100% completeness and <15% ill-defined codes) [27]. Thirdly, we found a steady percentage of deaths from non-specified causes (11.5%); this expected result [27, 35], the reasons for which should be explored in future national mortality studies, may have underestimated the number of CCCs’ deaths. Fourth, we dropped variables that might have been relevant to PoD, due to high (above 15%) levels of missing data. Fifth, by establishing a 17-years age-limit (focused on the national paediatric setting), we probably missed deaths from childhood CCCs occurring in adulthood, therefore not fully capturing their impact in the healthcare system. Sixth, PoD only has three categories in the Portuguese death certificate; however, to date Portugal has no paediatric hospices, so “domicile” can be assumed to represent home. If services become implemented, coding should expand to include “PPC unit/hospice”. Seventh, small differences and weak associations have statistical significance in a large dataset, hence we advise considering the magnitude of our findings. Still, only 5/30 sub-regions had less than 100 deaths from CCCs. Eighth and finally, when interpreting results for aggregated area level information ecological fallacy may occur, i.e. generalising area information to all individuals within. However, only one such variable (sub-region of residence) stayed in the final model.

## Conclusion

Our study provides recent and robust epidemiological data supporting that death from conditions with potential PPC needs is increasingly important in Paediatrics and occurring later in the disease trajectory. It also suggests that where PPC provision is lacking, children and families’ needs are probably largely unmet, as embodied by low and decreasing proportions of HD.

To revert this scenario, it is urgent to implement PPC services and ensure adequate home support for children, regardless of their age, condition and place of residence – factors for which we disclosed wide variations. These findings should be complemented with national surveys to understand needs and preferences of children with CCCs and their relatives, and by studies comparing care experiences in different settings.

## Additional files

Additional file 1: Table S1. ICD codes used to recode cause of death. (DOCX 93 kb)

Additional file 2: Figure S1. Trend for place of death of 0–17 year-old decedents in Portugal (1987–2011, *N* = 38,870). (DOCX 131 kb)

Additional file 3: Table S2. Annual trend for home death in 0–17 year-old decedents from complex chronic conditions in Portugal (1987–2011). (DOCX 65 kb)

Additional file 4: Figure S2. Trend for place of death of 0–17 year-old decedents from CCCs in Portugal (1987–2011, *N* = 10,571). (DOCX 119 kb)

Additional file 5: Figure S3. Trend for home death in 0–17 year-old decedents from complex chronic conditions in Portugal (1987–2011, N = 10,571) by age groups below and above 1 year. (DOCX 109 kb)

Additional file 6: Figure S4. Trend for home death in 0–17 year-old decedents from cancer and non-cancer CCCs in Portugal (1987–2011, N = 10,571). (DOCX 101 kb)

Additional file 7: Table S3. Bivariate analysis of factors associated with home death for 0–17 years-old decedents from complex chronic conditions in Portugal (1987–2011). (DOCX 73 kb)

Additional file 8: Figure S5. Percentage of deaths occurring at home, by subregion NUTS III, in 0–17 year-old decedents from CCCs in Portugal (1987–2011, N = 10,440). (DOCX 502 kb)

Additional file 9: Table S4. Bivariate analysis of parents’ factors associated with home death for <1yo decedents from complex chronic conditions in Portugal (1987–2011). (DOCX 56 kb)

## Abbreviations

CCC: Complex chronic conditions
HD: Home death
ICD: International Classification of Diseases
MVA: Multivariate analysis
NIS: National Institute of Statistics
OMC: Other medical causes
PC: Palliative care
PoD: Place of death
PPC: Paediatric palliative care
