# Supplementary material for: Trends in cause and place of death for children in Portugal (a European country with no Paediatric palliative care) during 1987–2011: a population-based study
Source: BMC Pediatr. 2017 Dec 22;17:215. doi: 10.1186/s12887-017-0970-1 (PMC5741889; doi:10.1186/s12887-017-0970-1)
Supplement: Supplementary file 1 — ICD codes used to recode cause of death. (DOCX 93 kb) [file 12887_2017_970_MOESM1_ESM.docx]

| **ADDITIONAL TABLE 1. ICD codes used to recode cause of death.** | | | | |
| --- | --- | --- | --- | --- |
| **Category** | **Sub-category** | | **ICD-9** | **ICD-10** |
| **Neuromuscular** | Brain and spinal cord malformations | | 7400-7429 | Q000-2, 010-9, 029, 039, 043, 048, 050-2, 054-7, 059, 062, 064, 068, 078, 079 |
|  | Mental retardation | | 3180-3182 | F719, 729, 739 |
|  | CNS degeneration and disease | | 3300-3309  3340-3342  3350-3359 | E-752, 754 / G109, 111, 114, 118, 120-2, 128-9, 20, 210-3, 218-9, 238, 241-5, 248, 250, 253, 255-6, 258-9, 309-11, 318-20, 328, 900, 908-11, 937, 950-1, 958-9, 990, 992 / M890 |
|  | Infantile cerebral palsy | | 3430-3439 | G801-2, 808-9 |
|  | Epilepsy | | 3450-3459 | G403, 410-2 |
|  | Muscular dystrophies and myopathies | | 3590-3593 | G710-2, 723 |
| **Cardiovascular** | Heart and great vessel malformations | | 7450-7474 | Q200-1, 203-5, 208-13, 220-1, 223-5, 230-4, 240, 242-6, 248-52, 254, 257, 262-3, 268-9 |
|  | Cardiomyopathies | | 4250-4254 / 4291 | I421, 423-4, 428, 515 |
|  | Conduction disorders and dysrhythmias | | 4260-4274, 4276-4279 | I440-3, 446-7, 451-6, 458-9, 471-2, 479, 480-9, 490-1, 494-5, 498-9 |
| **Respiratory** | Respiratory malformations | | 7480-7489 | Q300, 308, 310, 324, 330, 334, 336, 338-9, 348-9 |
|  | Chronic respiratory disease | | 7707 | P279 |
|  | Cystic fibrosis | | 2770 | E840-1, 848-9 |
| **Renal** | Congenital anomalies | | 7530-7539 | Q600, 610-5, 618-9, 623, 628, 638, 641, 643-4, 647, 649 |
|  | Chronic renal failure | | 5850-5859 | N189 |
| **Gastrointestinal** | Congenital anomalies | | 7503, 7511-7513, 7516-7519 | Q391, 419, 429, 431, 441-2, 445-6, 453, 458-9 |
|  | Chronic liver disease and cirrhosis | | 5714-5719 | K730, 738-9, 745-6, 760, 769 |
|  | Inflammatory bowel disease | | 5550-5569 | K500-1, 508-14, 518-9 |
| **Hematology and immunodeficiency** | Sickle cell disease | | 2825-2826 | D570-3 |
|  | Hereditary anaemias | | 2820-2824 | D551, 558, 569, 580-1 |
|  | Hereditary immunodeficiency | | 2790-2799, 2881-2882, 4461 | D719, 720, 800-5, 808, 814, 819-21, 830-1, 838, 849, 898-9 / M303, 359 |
|  | HIV disease | | 0420-0449 | B24 (currently B20 / B200-208; B249) |
| **Metabolic** | Aminoacid metabolism | 2700-2709 | | E700, 708, 710, 720-2, 728-9 |
|  | Carbohydrate metabolism | 2710-2719 | | E739-42, 748-9 |
|  | Lipid metabolism | 2720-2729 | | E756, 780-4, 786, 788-9, 881 |
|  | Storage disorders | 2773, 2775 | | E763, 806, 853, 858 |
|  | Other metabolic disorders | 2750-2753, 2772/4, 2776/8/9 | | E798, 806, 830-1, 833-4, 880, 888-9 |
|  |  |  | | *(cont.)* |
|  |  |  | |  |
| **ADDITIONAL TABLE 1. ICD codes used to recode cause of death (cont.).** | | | | |
| **Other congenital/genetic defect** | Chromosomal anomalies | 7580-7589 | | Q909, 913, 917, 930, 950, 952, 968, 984, 988, 999 |
|  | Bone and joint anomalies | 2594, 7373, 7560-7565 | | E343 / M410-3, 965 / Q750-3, 758-62, 764-6, 773, 776, 780-2, 788-9 |
|  | Diaphragm and abdominal wall | 5533, 7566-7567 | | K449 / Q791, 795 |
|  | Other congenital anomalies | 7597-7599 | | Q870-2, 874, 878, 897-9, 992 |
| **Malignancy** |  | 1400-2089, 2350-2399  Solid  1400-1999, 2350-2386, 2388-2399  Hematological  2000-2089, 2387 | | C000-D489, H350, N648, Q850  Solid  C000-C809 / C970-D459 / D480-D489  Hematological  C810-969 / D460-479 |
| **Trauma** |  | 8000-9999 | | S000-T889 / V000-Y999 |
| **Signs and symptoms not elsewhere classified** |  | 7800-7999 | | R00-R99 |
| **All other causes** |  | Remaining codes | | |
| CNS – Central Nervous System.  Source of ICD-9 codes: Feudtner C, Hays RM, Haynes G et al. Deaths attributed to pediatric complex chronic conditions: national trends and implications for supportive care services. Pediatrics 2001; 107: e99. doi: 10.1542/peds.107.6.e99.  Source of ICD-10 codes: Pousset G, Bilsen J, Cohen J et al. Deaths of children occurring at home in six European countries. Child Care Health Dev 2009; 36(3): 375-84. doi: 10.1111/j.1365-2214.2009.01028.x.   - Codes for trauma and hematological malignancy: [www.icd9data.com](http://www.icd9data.com), [www.icd10data.com](http://www.icd10data.com) | | | | |
