# Supplementary material for: Trends in cause and place of death for children in Portugal (a European country with no Paediatric palliative care) during 1987–2011: a population-based study
Source: BMC Pediatr. 2017 Dec 22;17:215. doi: 10.1186/s12887-017-0970-1 (PMC5741889; doi:10.1186/s12887-017-0970-1)
Supplement: Supplementary file 3 — Annual trend for home death in 0–17 year-old decedents from complex chronic conditions in Portugal (1987–2011). (DOCX 65 kb) [file 12887_2017_970_MOESM3_ESM.docx]

| **ADDITIONAL FILE 3: TABLE S2. Annual trend for home death in 0-17 year-old decedents from complex chronic conditions in Portugal (1987-2011).** | | | |
| --- | --- | --- | --- |
| **Year** | **All deaths (N)** | **Home deaths** | |
|  |  | **N** | **%** |
| **1987** | 773 | 275 | 35.6 |
| **1988** | 718 | 232 | 32.3 |
| **1989** | 686 | 205 | 29.9 |
| **1990** | 654 | 163 | 24.9 |
| **1991** | 659 | 176 | 26.7 |
| **1992** | 610 | 130 | 21.3 |
| **1993** | 593 | 119 | 20.1 |
| **1994** | 566 | 104 | 18.4 |
| **1995** | 517 | 99 | 19.1 |
| **1996** | 502 | 67 | 13.3 |
| **1997** | 447 | 74 | 16.6 |
| **1998** | 464 | 50 | 10.8 |
| **1999** | 413 | 58 | 14.0 |
| **2000** | 377 | 43 | 11.4 |
| **2001** | 351 | 31 | 8.8 |
| **2002** | 302 | 23 | 7.6 |
| **2003** | 272 | 39 | 14.3 |
| **2004** | 245 | 27 | 11.0 |
| **2005** | 231 | 21 | 9.1 |
| **2006** | 223 | 22 | 9.9 |
| **2007** | 229 | 18 | 7.9 |
| **2008** | 201 | 22 | 10.9 |
| **2009** | 181 | 16 | 8.8 |
| **2010** | 166 | 16 | 9.6 |
| **2011** | 191 | 22 | 11.5 |
| *Total* | *10571* | *2052* | *19.4* |
