# Supplementary material for: Trends in cause and place of death for children in Portugal (a European country with no Paediatric palliative care) during 1987–2011: a population-based study
Source: BMC Pediatr. 2017 Dec 22;17:215. doi: 10.1186/s12887-017-0970-1 (PMC5741889; doi:10.1186/s12887-017-0970-1)
Supplement: Supplementary file 4 — Trend for place of death of 0–17 year-old decedents from CCCs in Portugal (1987–2011, N = 10,571). (DOCX 119 kb) [file 12887_2017_970_MOESM4_ESM.docx]

**ADDITIONAL FIGURE 2. Trend for place of death of 0-17 year-old decedents from CCCs in Portugal (1987-2011, N=10571).**

“Elsewhere” represents hospital (79.3%) and other places (1.3%). Dashed lines represent age and gender directly standardised percentages, taking as standard the decedent population of the first year, 1987. λ^2^ for trend (home vs. elsewhere) 406.900, 1df, p<0.001.
