# Supplementary material for: Trends in cause and place of death for children in Portugal (a European country with no Paediatric palliative care) during 1987–2011: a population-based study
Source: BMC Pediatr. 2017 Dec 22;17:215. doi: 10.1186/s12887-017-0970-1 (PMC5741889; doi:10.1186/s12887-017-0970-1)
Supplement: Supplementary file 5 — Trend for home death in 0–17 year-old decedents from complex chronic conditions in Portugal (1987–2011, N = 10,571) by age groups below and above 1 year. (DOCX 109 kb) [file 12887_2017_970_MOESM5_ESM.docx]

**ADDITIONAL FIGURE 3. Trend for home death in 0-17 year-old decedents from complex chronic conditions in Portugal (1987-2011, N=10571) by age groups below and above 1 year.**

λ^2^ for trend (home vs. elsewhere): <1y 175.561, 1df, p<0.001; ≥1y 365.356, 1df, p<0.001.
