# Supplementary material for: Trends in cause and place of death for children in Portugal (a European country with no Paediatric palliative care) during 1987–2011: a population-based study
Source: BMC Pediatr. 2017 Dec 22;17:215. doi: 10.1186/s12887-017-0970-1 (PMC5741889; doi:10.1186/s12887-017-0970-1)
Supplement: Supplementary file 6 — Trend for home death in 0–17 year-old decedents from cancer and non-cancer CCCs in Portugal (1987–2011, N = 10,571). (DOCX 101 kb) [file 12887_2017_970_MOESM6_ESM.docx]

** ADDITIONAL FIGURE 4. Trend for home death in 0-17 year-old decedents from cancer and non-cancer CCCs in Portugal (1987-2011, N=10571).**

λ^2^ for trend (home vs. elsewhere): cancer 308.630, 1df, p<0.001; non-cancer 166.615, 1df, p<0.001.
