# Supplementary material for: Trends in cause and place of death for children in Portugal (a European country with no Paediatric palliative care) during 1987–2011: a population-based study
Source: BMC Pediatr. 2017 Dec 22;17:215. doi: 10.1186/s12887-017-0970-1 (PMC5741889; doi:10.1186/s12887-017-0970-1)
Supplement: Supplementary file 7 — Bivariate analysis of factors associated with home death for 0–17 years-old decedents from complex chronic conditions in Portugal (1987–2011). (DOCX 73 kb) [file 12887_2017_970_MOESM7_ESM.docx]

| **ADDITIONAL FILE 3: TABLE S3. Bivariate analysis of factors associated with home death for 0-17 years-old decedents from complex chronic conditions in Portugal (1987-2011).** | | | | |  |
| --- | --- | --- | --- | --- | --- |
| **Variable** | **Category** | **Home death** | | |  |
|  |  | **N** | **%** | **p-value (df)** | |
| **Gender (N=10570)** | Male | 1085 | 18.7 | 0.031 (1)^a^ | |
|  | Female | 967 | 20.3 |  | |
| **Age (N=10571)** | 0-27 days | 101 | 3.3 | <0.001 (5)^b^ | |
|  | 28-364 days | 415 | 18.1 |  | |
|  | 1-5 years | 497 | 26.8 |  | |
|  | 6-10 years | 430 | 34.2 |  | |
|  | 11-14 years | 314 | 30.0 |  | |
|  | 15-17 years | 295 | 27.6 |  | |
| **Nationality (N=9180)** | Portuguese | 1815 | 25.5 | <0.001 (1)^a^ | |
|  | Other | 212 | 10.1 |  | |
| **Diagnosis (N=10571)** | Cancer | 794 | 28.2 | <0.001 (8) | |
|  | Neuromuscular | 623 | 29.3 |  | |
|  | Cardiovascular | 333 | 12.8 |  | |
|  | Respiratory | 21 | 5.0 |  | |
|  | Renal | 24 | 12.3 |  | |
|  | Gastro-intestinal | 23 | 9.5 |  | |
|  | Hematology & Immunodeficiency | 21 | 11.6 |  | |
|  | Metabolic | 55 | 19.4 |  | |
|  | Other congenital & genetic | 158 | 9.2 |  | |
| **Population density (N=10440)** | 5^th^ quintile (most populated) | 724 | 14.2 | <0.001 (4)^b^ | |
|  | 4^th^ quintile | 569 | 26.1 |  | |
|  | 3^rd^ quintile | 331 | 20.0 |  | |
|  | 2^nd^ quintile | 328 | 34.7 |  | |
|  | 1^st^ quintile (least populated) | 94 | 17.1 |  | |
| **Bed ratio (N=10440)** | Above average | 329 | 9.9 | <0.001 (1)^a^ | |
|  | Below average | 1717 | 24.1 |  | |
| **Semester of death (N=10571)** | April-September | 905 | 18.0 | <0.001^c^ | |
|  | October-March | 1147 | 20.7 |  | |
| df – degrees of freedom; ^a^ **λ^2^** test; ^b^ Kruskal Wallis test; ^c^ **λ^2^** test for trend  The fifth population density quintile was the most populated. National bed ratio average (practiced allotment in health establishments) was 3.4 beds per 1000 inhabitants in 2011 and 3.7 in 2002; there was no change in regional categorization between these time-points. | | | | |  |
