# Supplementary material for: Trends in cause and place of death for children in Portugal (a European country with no Paediatric palliative care) during 1987–2011: a population-based study
Source: BMC Pediatr. 2017 Dec 22;17:215. doi: 10.1186/s12887-017-0970-1 (PMC5741889; doi:10.1186/s12887-017-0970-1)
Supplement: Supplementary file 8 — Percentage of deaths occurring at home, by subregion NUTS III, in 0–17 year-old decedents from CCCs in Portugal (1987–2011, N = 10,440). (DOCX 502 kb) [file 12887_2017_970_MOESM8_ESM.docx]

**ADDITIONAL FIGURE 5. Percentage of deaths occurring at home, by subregion NUTS III, in 0-17 year-old decedents from CCCs in Portugal (1987-2011, N=10440).**

λ^2^ 615.799, 29df, p<0.001.
