# Supplementary material for: Trends in cause and place of death for children in Portugal (a European country with no Paediatric palliative care) during 1987–2011: a population-based study
Source: BMC Pediatr. 2017 Dec 22;17:215. doi: 10.1186/s12887-017-0970-1 (PMC5741889; doi:10.1186/s12887-017-0970-1)
Supplement: Supplementary file 9 — Bivariate analysis of parents’ factors associated with home death for <1yo decedents from complex chronic conditions in Portugal (1987–2011). (DOCX 56 kb) [file 12887_2017_970_MOESM9_ESM.docx]

| **ADDITIONAL FILE 4: TABLE S4. Bivariate analysis of parents’ factors associated with home death for <1yo decedents from complex chronic conditions in Portugal (1987-2011).** | | | | |
| --- | --- | --- | --- | --- |
| **Variable** | **Category** | **Home death** | | |
|  |  | **N** | **%** | **p-value (df)** |
| **Mother’s educational level (N=5180)** | Illiterate / Primary | 435 | 10.9 | <0.001 (1)^b^ |
|  | Secondary / Higher | 59 | 5.0 |  |
| **Mother’s age (N=5209)** | <20 years | 33 | 9.4 | 0.640 (1)^a^ |
|  | 20-29 years | 284 | 10.0 |  |
|  | 30-39 years | 151 | 8.4 |  |
|  | >40 years | 28 | 13.0 |  |
| **Father’s age (N=4858)** | <20 years | 3 | 3.9 | 0.888 (1)^a^ |
|  | 20-29 years | 223 | 10.2 |  |
|  | 30-39 years | 181 | 8.5 |  |
|  | >40 years | 45 | 9.8 |  |
| **Father’s working status (N=4849)** | Employed | 424 | 9.2 | <0.001 (3)^b^ |
|  | Unemployed | 9 | 9.3 |  |
|  | Not active | 5 | 6.0 |  |
|  | Other | 16 | 29.1 |  |
| df – degrees of freedom; ^a^ Kruskal-Wallis test; ^b^ λ^2^ test | | | | |
